# A Thalamocortical Neural Mass Model of the EEG during NREM Sleep and Its Response to Auditory Stimulation

**DOI:** 10.1371/journal.pcbi.1005022

**Published:** 2016-09-01

**Authors:** Michael Schellenberger Costa, Arne Weigenand, Hong-Viet V. Ngo, Lisa Marshall, Jan Born, Thomas Martinetz, Jens Christian Claussen

**Affiliations:** 1 Institute for Neuro- and Bioinformatics, University of Lübeck, Lübeck, Germany; 2 Institute for Medical Psychology and Behavioral Neurobiology, University of Tübingen, Tübingen, Germany; 3 Graduate School for Computing in Medicine and Life Science, University of Lübeck, Lübeck, Germany; 4 Institute of Experimental and Clinical Pharmacology and Toxicology, University of Lübeck, Lübeck, Germany; 5 Center for Integrative Neuroscience, University of Tübingen, Tübingen, Germany; 6 Computational Systems Biology, Jacobs University Bremen, Bremen, Germany; Brain and Spine Institute (ICM), FRANCE

## Abstract

Few models exist that accurately reproduce the complex rhythms of the thalamocortical system that are apparent in measured scalp EEG and at the same time, are suitable for large-scale simulations of brain activity. Here, we present a neural mass model of the thalamocortical system during natural non-REM sleep, which is able to generate fast sleep spindles (12–15 Hz), slow oscillations (<1 Hz) and K-complexes, as well as their distinct temporal relations, and response to auditory stimuli. We show that with the inclusion of detailed calcium currents, the thalamic neural mass model is able to generate different firing modes, and validate the model with EEG-data from a recent sleep study in humans, where closed-loop auditory stimulation was applied. The model output relates directly to the EEG, which makes it a useful basis to develop new stimulation protocols.

## Author Summary

Sleep plays a pivotal role for the consolidation of memory. While REM sleep had originally been the focus of research due to its similarity with wakefulness, more recent studies suggest that different sleep stages are responsible for the consolidation of different types of memory. To better understand the changes in neuronal dynamics between the different sleep stages, neural mass models are a valuable tool, as they relate directly to the large-scale dynamics measured by an EEG. Here, we present a model of the sleeping thalamocortical system. We first show that the isolated thalamic submodule is able to generate different oscillatory behavior found in vivo. Furthermore, the full thalamocortical model reproduces the EEG of sleep stages N2 and N3 and preserves the temporal relationship between cortical K-complexes/slow oscillations and thalamic sleep spindles. A comparison with event related potential data from a recent sleep study in humans demonstrates its possible application in predicting the outcome of external stimulation on EEG rhythms. Our study shows, that a neural mass model incorporating few key mechanisms is sufficient to reproduce the complex brain dynamics observed during sleep.

## Introduction

In the human electroencephalogram (EEG) the most prominent features of non-rapid eye movement (NREM) sleep are neocortical slow oscillations (SO) at ∼ 0.8 Hz [1] and fast thalamocortical spindles characterized by a waxing and waning waveform, with a frequency of 12 to 15 Hz [2]. The cellular basis of the slow oscillation is a widespread alternation of activity in neocortical networks between an active (up) state and a hyperpolarized silent (down) state [2–4]. Fast sleep spindles are generated by the interaction of inhibitory reticular thalamic (RE) and excitatory thalamocortical (TC) neurons [5] and preferentially occur during the up state of SOs [6].

Many studies indicate a functional role of slow wave sleep (SWS) in the formation of memory [7, 8]. The synchronization of fast spindle activity to the depolarized up state is mediated by the thalamocortical loop. It appears to be critical for consolidation as it provides a window of opportunity and favorable conditions for plastic changes [9–11]. It has been shown that memory consolidation can be improved by transcranial electric and auditory stimulation [12, 13]. Auditory stimulation in synchrony with the brain’s own rhythm turned out to be particularly effective.

Detailed knowledge of how different stimulation modalities effect critical brain rhythms as well as the ramifications of potential stimulation parameters on their efficacy would enable an optimization of stimulation protocols and consequently an advantage for experiments in basic research and clinical applications. Mathematical models and computational approaches can yield meaningful insights into the underlying dynamics as well as provide predictions for further experiments. Multiple models based on networks of single cells have addressed brain activity during sleep in the cortex [14], thalamus [15–17], as well as the coupled thalamocortical system [18–21]. However, for investigations of macroscopic phenomena these models have disadvantages due to their complexity and computational load.

Neural mass models have shown great success in elucidating the generation of brain rhythms and evoked responses of the awake brain [22–25]. They describe the dynamics of a large number of cells by the evolution of a single population average and provide an output which directly relates to EEG signals [26, 27]. By still allowing an integration of physiologically motivated cell dynamics via intrinsic currents, neural mass models represent a compromise between a very detailed and an abstract modeling approach and provide insights into the dynamic repertoire of the neural populations via bifurcation analysis [28–30].

In previous work, we have shown that a cortical neural mass model equipped with an additive activity-dependent feedback current can generate a time series that closely resembles the EEG signal of sleep stages N2 and N3, without spindles [30]. Here, we extend the cortical model by adding a thalamic module to incorporate spindle activity and investigate the underlying dynamics of the coupled system. We test the evocability of SOs and spindles by auditory stimulation during NREM sleep and validate our results with scalp EEG data from a recent sleep study in humans [13]. Our findings add further support to the dynamic mechanisms proposed in [30].

## Methods

In the following sections we detail the thalamocortical model. First we give a brief overview of the concept of neural mass models. We then introduce the cortical and thalamic modules and motivate the extensions with respect to sleep. The full equations of the thalamocortical model are given in S1 Text.

### Neural mass framework

The conductance based neural mass model employed here is derived from [31–34]. Instead of considering the evolution of high-dimensional states in a large ensemble of single neurons, the population activity can be approximated by the evolution of the mean membrane voltage of that population. The complex spiking dynamics is replaced by an empirical firing rate function

$$\begin{array}{c} Q_{k}=\frac{Q_{k}^{\text{max}}}{1+\exp(-\left(V_{k}-\theta \right)/\sigma_{k})}, \end{array}$$ (1)

with maximal firing rate $Q_{k}^{\text{max}}$, firing threshold *θ* and neural gain *σ*_*k*_. It converts the average membrane voltage *V*_*k*_ of the population *k* to an output spike rate. Here, *k* ∈ {*p*, *i*, *t*, *r*} stands for cortical pyramidal, cortical interneuron, thalamic relay and thalamic reticular populations, respectively. The firing rate function often has a sigmoidal shape and can be interpreted as stemming from the fluctuations of neuronal states or a distribution of thresholds in the population [35].

The spike rate *Q*_*k*′_ of the presynaptic population *k*′ then elicits a postsynaptic response *s*_*mk*_ within the receiving population *k*. The strength of this response can be calculated by the convolution

$$s_{mk}=\sum_{k^{\prime }}\alpha_{m}⊗N_{kk^{\prime }}Q_{k^{\prime }},$$ (2)

of the incoming spike rate *Q*_*k*′_, scaled with the averaged connectivity constant *N*_*kk*′_ between the presynaptic population *k*′ and the postsynaptic population *k* and an alpha function

$$\alpha_{m}=\gamma_{m}^{2}t\exp\left(-\gamma_{m}t\right),$$ (3)

representing the average synaptic response to a single spike. Here, *γ*_*m*_ depicts the rate constant of the synaptic response, whereas *m* ∈ {*e*, *g*, *r*} denotes the type of synapse with *e* standing for excitatory AMPA and *g*, *r* for inhibitory GABA synapses in the cortex and thalamus, respectively.

The evolution of the population membrane voltage *V*_*k*_ obeys

$$\begin{array}{ll} \tau_{k}{\dot{V}}_{k} & =-(V_{k}-E_{\text{L}}^{k})-w_{\text{AMPA}}s_{ek}(V_{k}-E_{\text{AMPA}})-w_{\text{GABA}}s_{gk}(V_{k}-E_{\text{GABA}}),\\ & =-J_{\text{L}}-J_{\text{AMPA}}(s_{ek})-J_{\text{GABA}}(s_{gk}), \end{array}$$ (4)

where *w* denotes a synaptic input rate that scales the input *s*_*mk*_ and *E* the corresponding Nernst potential. While we lean on the naming convention of Hodgkin-Huxley models to highlight the structural similarity, please note that the above quantities *J* and *w* have different units. For the sake of simplicity we have normalized the synaptic rates *w* to 1, absorbing their numerical values into the connectivities *N*_*kk*′_.

Intrinsic currents may additionally be included in the equation of the mean membrane voltage, given their time constant is large compared to the time constant of neuronal spiking [35, 36]. To emphasize the connection to physiology and to better differentiate between the core neural mass model and the additional mechanisms we will denote them with *I* and introduce a capacitive coupling via to the neural mass via the membrane capacity *C*_*m*_.

The signal measured in the EEG stems mainly from the activity of pyramidal neurons [37]. We use the pyramidal membrane voltage as our model output, which is similar to other studies [38, 39]. Populations comprising multiple clusters have been considered in [40] and lead to interesting effects. In order to keep the complexity of the model low we consider a single point source. Therefore, filtering effects by the skull/scalp can be approximated by a linear scaling and do not affect the interaction between thalamus and cortex.

### Cortex

We use the neural mass model described in [30], which captures essential features of the EEG during NREM sleep. In brief, it consists of an excitatory and an inhibitory neural mass, representing populations of pyramidal neurons (p) and interneurons (i), respectively. The pyramidal population contains a slow, additive and activity-dependent firing rate adaptation,

$$\begin{array}{cc} \tau_{\text{Na}}\left[\dot{\text{Na}}\right] & =\alpha_{\text{Na}}Q_{p}\left(V_{p}\right)-{\text{Na}}_{\text{pump}}\left(\left[\text{Na}\right]\right),\\ I_{\text{KNa}} & ={\bar{g}}_{\text{KNa}}w_{k}\left(\left[\text{Na}\right]\right)\left(V_{p}-E_{K}\right), \end{array}$$ (5)

which is believed to be the main driver for the transition to the silent (down) state, while the active (up) state is maintained by synaptic transmission [3, 14, 41].

The membrane potentials evolve according to

$$\begin{array}{cc} \tau_{p}{\dot{V}}_{p} & =-J_{L}^{p}-J_{\text{AMPA}}\left(s_{ep}\right)-J_{\text{GABA}}\left(s_{gp}\right)-C_{m}^{-1}\tau_{p}I_{\text{KNa}},\\ \tau_{i}{\dot{V}}_{i} & =-J_{L}^{i}-J_{\text{AMPA}}\left(s_{ei}\right)-J_{\text{GABA}}\left(s_{gi}\right). \end{array}$$ (6)

and are linked by the AMPA and GABAergic synaptic inputs.

The transition from N2 to N3 is achieved by changing the neural gain, *σ*_*p*_, and the strength of the adaptation, given by ${\bar{g}}_{\text{KNa}}$. Both parameters are known to be influenced by acetylcholine, serotonin, norepinephrine and dopamine [42–52], whose levels change throughout the sleep-wake cycle [53]. For consistency, we maintained the original model, adjusting only the excitatory connection strengths to compensate for additional input from the thalamic module.

### Thalamus

The thalamic module comprises similarly an excitatory and an inhibitory neural mass, representing a thalamocortical (t) and the reticular (r) nucleus. As in the cortical module module they are coupled via AMPA and GABA synapses but have different synaptic time constants and only the RE population possesses a self-connection.

Both populations are equipped with additional currents. The inclusion of those currents within the thalamic submodule is necessary because spindle oscillations require rebound bursts. In classical neural mass models, this kind of bursting is not possible due to the monotonic firing rate function and demands the inclusion of additional mechanisms. The same argument was used in a previous neural mass model of spindle activity [54] and a thalamocortical neural mass model of epileptic activity [55]. Finally, in [40] the authors arrive at a HH-type extension of their population model of thalamic burst activity, which has been derived from integrate-and-fire-or-burst neurons. The potassium leak current is given by

$$I_{\text{LK}}={\bar{g}}_{\text{LK}}\left(V_{k}-E_{K}\right),$$ (7)

as well as T-type calcium currents

$$I_{T}={\bar{g}}_{T}m_{\infty }^{2}h\left(V_{k}-E_{\text{Ca}}\right),$$ (8)

which deinactivate upon hyperpolarization. They are essential for the generation of low-threshold spikes (LTSs) and rebound bursts. We use the description of *I*_T_ given in [56] for the RE and the one in [16] for the TC population.

The TC population further includes the anomalous rectifier current

$$I_{h}={\bar{g}}_{h}\left(m_{h1}+g_{inc}m_{h2}\right)\left(V_{t}-E_{h}\right),$$ (9)

responsible for the waxing and waning structure of spindle oscillations in the isolated thalamus [15]. Other currents, such as the calcium-dependent potassium currents *I*_KCa_ and *I*_CAN_, are also known to play a role in spindle oscillations, but are omitted for simplicity. The thalamic module is summarized by

$$\begin{array}{cc} \tau_{t}{\dot{V}}_{t} & =-J_{L}^{t}-J_{\text{AMPA}}\left(s_{et}\right)-J_{\text{GABA}}\left(s_{rt}\right)-C_{m}^{-1}\tau_{t}\left(I_{\text{LK}}^{t}-I_{T}^{t}-I_{h}\right),\\ \tau_{r}{\dot{V}}_{r} & =-J_{L}^{r}-J_{\text{AMPA}}\left(s_{er}\right)-J_{\text{GABA}}\left(s_{rr}\right)-C_{m}^{-1}\tau_{r}\left(I_{\text{LK}}^{r}-I_{T}^{r}\right). \end{array}$$ (10)

Parameter settings for the currents are identical to [57], with the exception of the deactivation function $h_{\infty }^{t}$ of the thalamic relay population, that is shifted towards more depolarized membrane voltages.

### Full model

**Fig 1:**
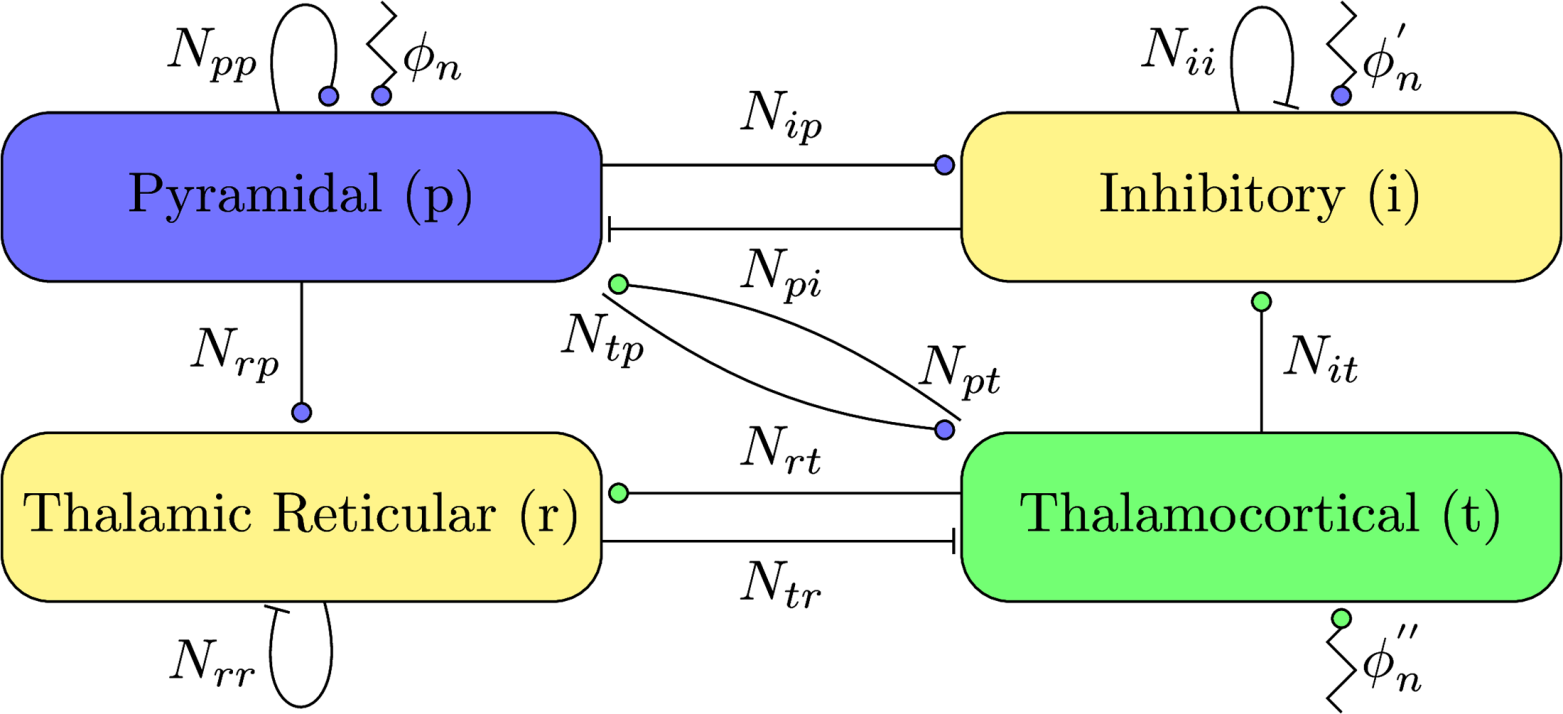
Connectivity of the thalamocortical model. Excitatory synapses are depicted by filled circles, inhibitory synapses by bars. Independent background noise entering the different populations is denoted by *ϕ*_*n*_, $\phi_{n}^{{}^{\prime }}$ and $\phi_{n}^{{}^{\prime \prime }}$, respectively. Stimulation is applied as an elevation in the mean of the background noise $\phi_{n}^{{}^{\prime \prime }}$ of the thalamic relay population.

The model consists of one thalamic and one cortical module. We assume the long range afferents from the cortical pyramidal population project to both populations of the thalamic nuclei, and the long range afferents of the thalamic relay population project to both populations of the cortex, as depicted in Fig 1. The delays introduced by these long range afferents might play a crucial role in cortical dynamics [58, 59]. However, as the axonal conduction delay between thalamus and cortex is rather small [60–63], we approximate it by a convolution with an alpha function [64], which can be written as

$${\ddot{\phi }}_{k}=\nu^{2}\left(Q_{k}\left(V_{k}\right)-\phi_{k}\right)-2\nu {\dot{\phi }}_{k},$$ (11)

where *ϕ*_*k*_ is the resulting delayed firing rate and *ν* depicts the axonal rate constant of that connection. We have provided a justification for that approximation in the supporting information S2 Text. In the case of short range connections *ϕ*_*k*_ can be replaced with *Q*_*k*_. The parameters of the full model are given in S1 Table.

### Auditory stimulation

We model an auditory stimulus as an elevation in background noise $\phi_{n}^{{}^{\prime \prime }}$ (square pulse) being gated through the thalamus. For all stimuli, we use a duration of 80 ms and 70 spikes per second.

### Event triggered averages

KCs and SOs were detected similar to [10]. The model output was bandpass filtered between 0.25 and 4 Hz. Zero-crossings were detected to extract the negative half-waves. Negative half-waves with peaks below -69 mV were considered to be KCs/SOs.

### Experimental data

Experimental data has been described in [65]. 11 Subjects were measured during two experimental nights in balanced order in a stimulation and control condition. For averages of the endogenous activity, data was taken from the control condition. See S1 Dataset.

### Computational methods

The model was implemented in C++ and run within MATLAB R2015b, using a stochastic Runge-Kutta method of 4th order [66] with a step size of 0.1 ms. The code can be found at github [67, 68].

## Results

In the following section we perform a bifurcation analysis and demonstrate the ability of the thalamic module to generate spindle oscillations and reproduce different experimental observations. Afterwards we investigate the interplay between thalamus and cortex to reproduce the characteristics of different sleep stages. Finally, we examine the effect of auditory stimulation in the model and compare different stimulation protocols with experimental findings.

### Thalamic spindle oscillations

In the isolated thalamic module, incorporation of the intrinsic currents may lead to oscillations in the spindle band. We follow closely the mechanisms established in the models by [15, 19, 20]. Physiologically, these oscillations are generated, through reciprocal interaction of the RE and TC populations. A LTS in the RE population causes hyperpolarization in the TC population, that deinactivates its T-type calcium current. Upon release from inhibition a rebound of activity occurs, that in turn drives the RE module to produce another LTS. Additionally, the deinactivation of the T-type calcium currents requires a strong tonic hyperpolarization by a potassium leak current [15, 19].

As previously shown in [5, 15, 20, 69], the rhythmicity of spindle occurrence and the waxing and waning of the spindle amplitude is caused by an anomalous rectifier channel *I*_h_. A sequence of LTS leads to the build-up of calcium, which increases the effective conductivity ${\bar{g}}_{h}={\bar{g}}_{h}\left(m_{h1}+g_{inc}m_{h2}\right)$ of *I*_h_. The ensuing depolarization of the TC population increasingly counteracts its ability to produce a LTS and terminates the spindle oscillation [70, 71].

**Fig 2:**
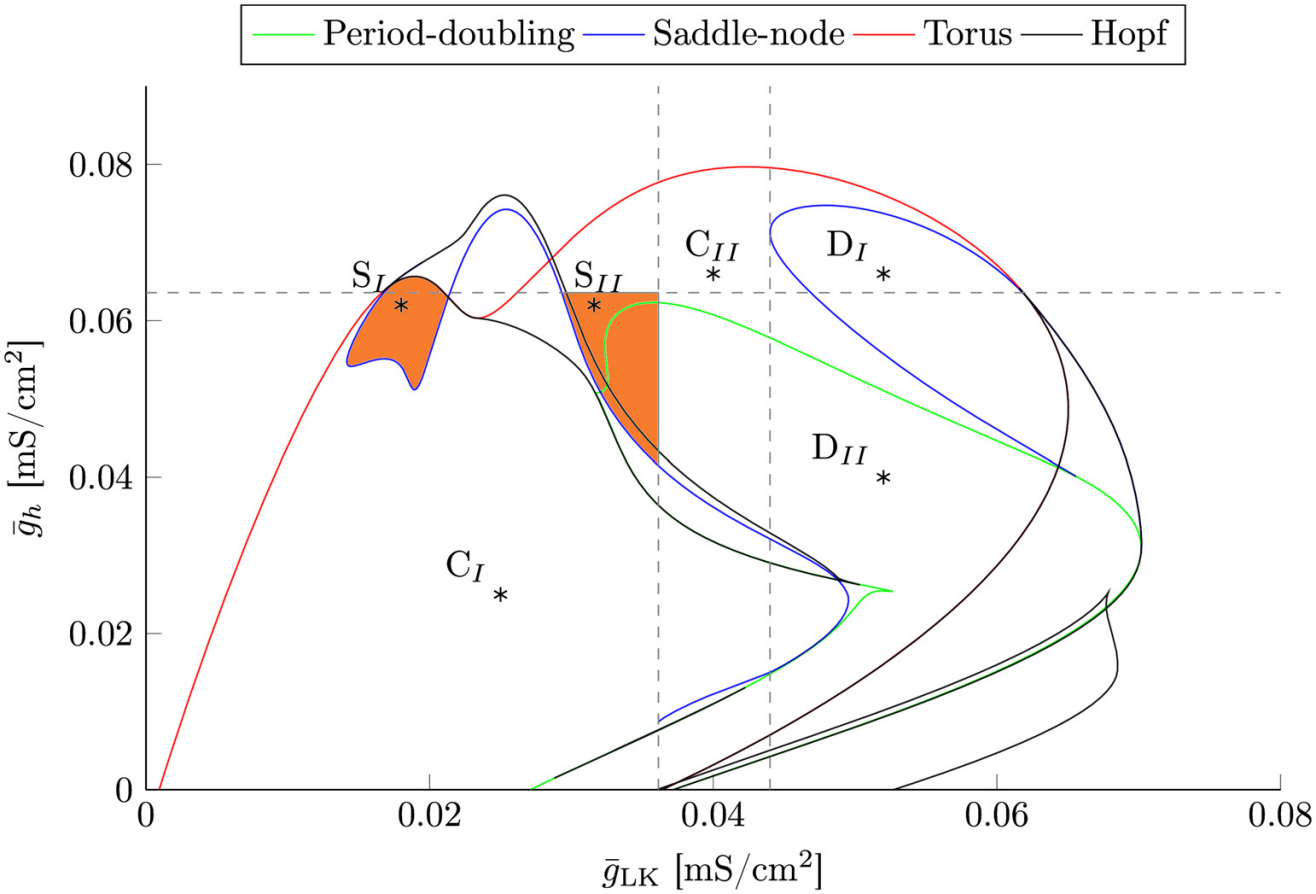
Two-dimensional bifurcation analysis. Here, we illustrate the bifurcation diagram of the isolated thalamus with respect to the two key parameters ${\bar{g}}_{h}$ and ${\bar{g}}_{\text{LK}}$. The interaction between the currents incorporated into the thalamic module results in the emergence of two torus bifurcations via a blue sky catastrophe. They lead to spindle oscillations in the orange shaded regions. The left spindle regime (S_I_) is encased by a Hopf and a torus bifurcation, whereas the right spindle regime (S_II_) is constrained by two global bifurcations that are indicated by the dashed gray lines. The vertical line marks the emergence of the torus bifurcation, whereas the horizontal gray line marks the cusp bifurcation where the two saddle-nodes that accompany the left torus bifurcation vanish. The torus bifurcation on the right marks the transition from spindle oscillations to delta oscillations. The labeled points mark the parameter settings utilized in Fig 3, which are given in Table 1.

**Fig 3:**
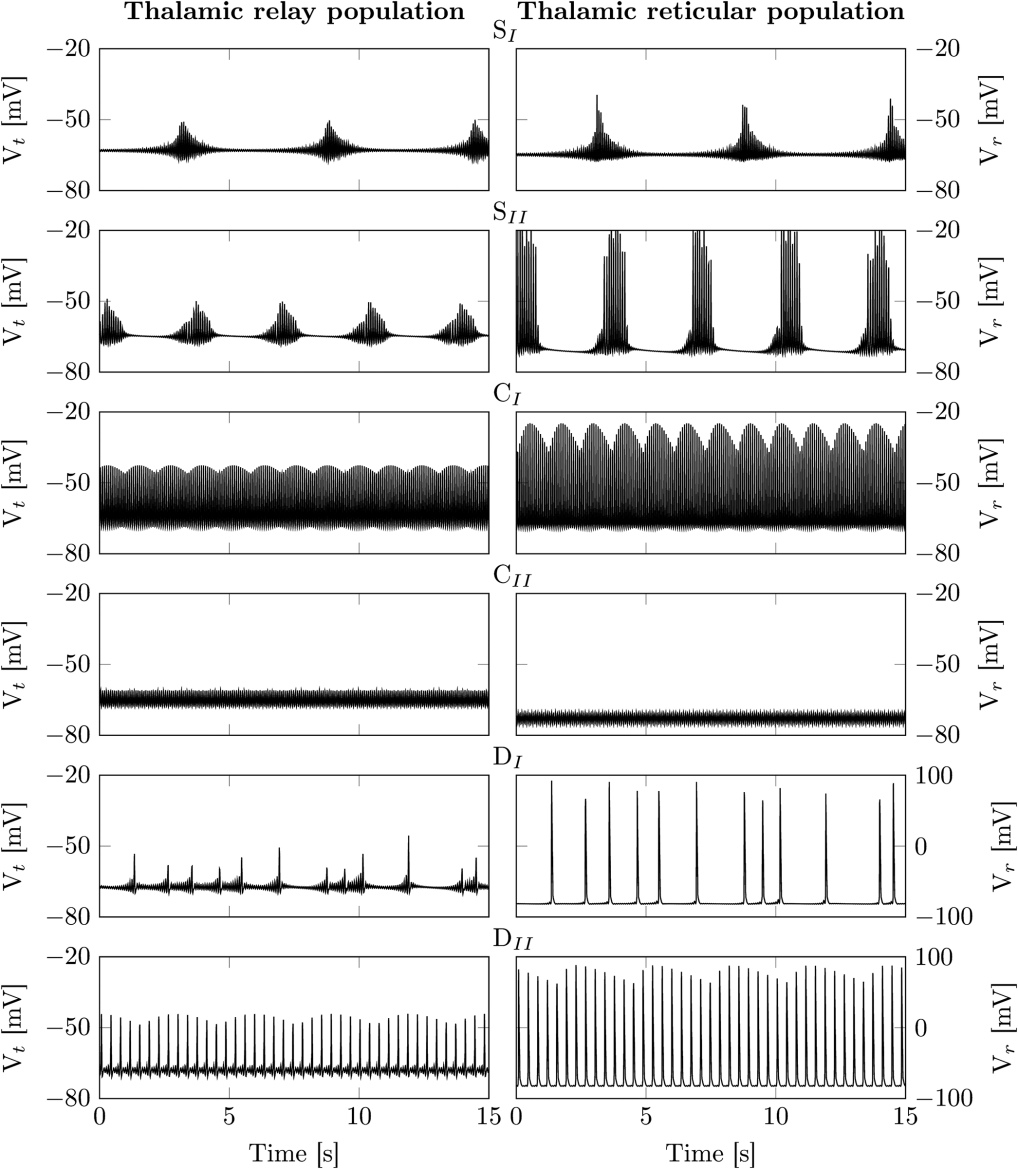
Dynamic modes of the isolated thalamic module. Here, we illustrate the different dynamic modes the isolated thalamic module exhibits. The left panels depict the thalamic relay membrane voltage, whereas the right panels illustrate that of the thalamic reticular population. The parameter values are depicted in Fig 2 and given in Table 1. S_I_ and S_II_: The isolated thalamus generates rhythmic spindle oscillations via a balanced interplay between *I*_T_ and *I*_h_. The length and the average time between spindles is governed by ${\bar{g}}_{h}$. C_I_ and C_II_: Outside of the spindle regime fast oscillations generated by the T-type calcium currents dominate and *I*_h_ is unable to sufficiently depolarize the thalamic relay population to cease them. D_I_ and D_II_: For strong hyperpolarization through *I*_LK_ the thalamic module switches into low frequency delta oscillations.

Therefore, we chose ${\bar{g}}_{h}$ and ${\bar{g}}_{\text{LK}}$ as bifurcation parameters. A two-dimensional bifurcation analysis of the thalamic module reveals the existence of a Hopf bifurcation, as depicted in Fig 2, which generates continuous oscillations in the spindle band due to hyperpolarization induced rebound bursts, see Fig 3-C_I_ and 3-C_II_ for representative time series.

**Table 1:** Parameter settings for the isolated thalamus.

Symbol	S_I_	S_II_	D_I_	D_II_	C_I_	C_II_	Unit
${\bar{g}}_{\text{LK}}$	0.018	0.032	0.052	0.052	0.025	0.04	mS/cm^2^
${\bar{g}}_{h}$	0.062	0.062	0.066	0.04	0.025	0.066	mS/cm^2^

Parameter settings of the isolated thalamus. This table lists the parameter values for the different dynamic regimes of the isolated thalamic module, that are utilized in Fig 3.

The torus bifurcations emerge from a blue sky catastrophe that is generated by the slow-fast interaction between the fast T-type channels and their slow modulation via *I*_h_, which is similar to other models that exhibit switching between tonic spiking and structured bursting activity [72, 73].

As depicted in Fig 3-S_I_ and 3-S_II_ this leads to spindle like oscillations in the orange shaded regions in Fig 2. The spindles exhibit an oscillation frequency of around 13 Hz. The spindle frequency depends on the strength of the T-type calcium current ${\bar{g}}_{T}$. Importantly, spindle oscillations are initiated intrinsically. The thalamic module does *not* require modulatory input from external sources to initiate/terminate them.

Activation of *I*_h_ is responsible for a refractory period that follows a spindle. As long as *I*_h_ activation persists, LTS generation is impeded and stronger perturbations are necessary to trigger spindle oscillations. Consequently, an increase in ${\bar{g}}_{h}$ results in a larger inter-spindle interval. The left spindle regime (S_I_) is encased by the Hopf and the torus bifurcation, whereas the right spindle regime (S_II_) is constrained by two global bifurcations that are indicated by the dashed gray lines. The vertical line marks the emergence of the torus bifurcation, whereas the horizontal gray line marks the cusp bifurcation where the two saddle-nodes that accompany the left torus bifurcation vanish.

Furthermore, for larger values of ${\bar{g}}_{\text{LK}}$ the model transitions from high frequency spindle oscillations to low frequency delta oscillations, e.g. Fig 3-D_I_ and 3-D_II_.

### K-complexes and spindles during sleep stage N2

In the coupled system, the cortex provides excitatory drive to the thalamic module, since it is predominantly in the active state. In order for the thalamic module to exhibit rhythmically occurring spindle oscillations we had to adjust ${\bar{g}}_{h}$ and ${\bar{g}}_{\text{LK}}$ slightly to account for that additional depolarization (see Table 2). We choose the right spindle regime, as it was suitable for reproduction of both sleep stage N2 and N3.

**Table 2:** TC parameter settings.

Symbol	N2	N3	Unit	Description
*σ*_*p*_	4.7	6	mV	Neuronal gain
${\bar{g}}_{\text{KNa}}$	1.33	1.88	mS/cm^2^	Adaption strength
${\bar{g}}_{\text{LK}}$	0.034	0.034	mS/cm^2^	Potassium leak conductance
${\bar{g}}_{h}$	0.052	0.062	mS/cm^2^	*h*-current conductance

Parameter settings of the full model. This table shows the different parameter settings of the full model used throughout this study. Columns N2 and N3 give the parameters for the respective sleep stages.

**Fig 4:**
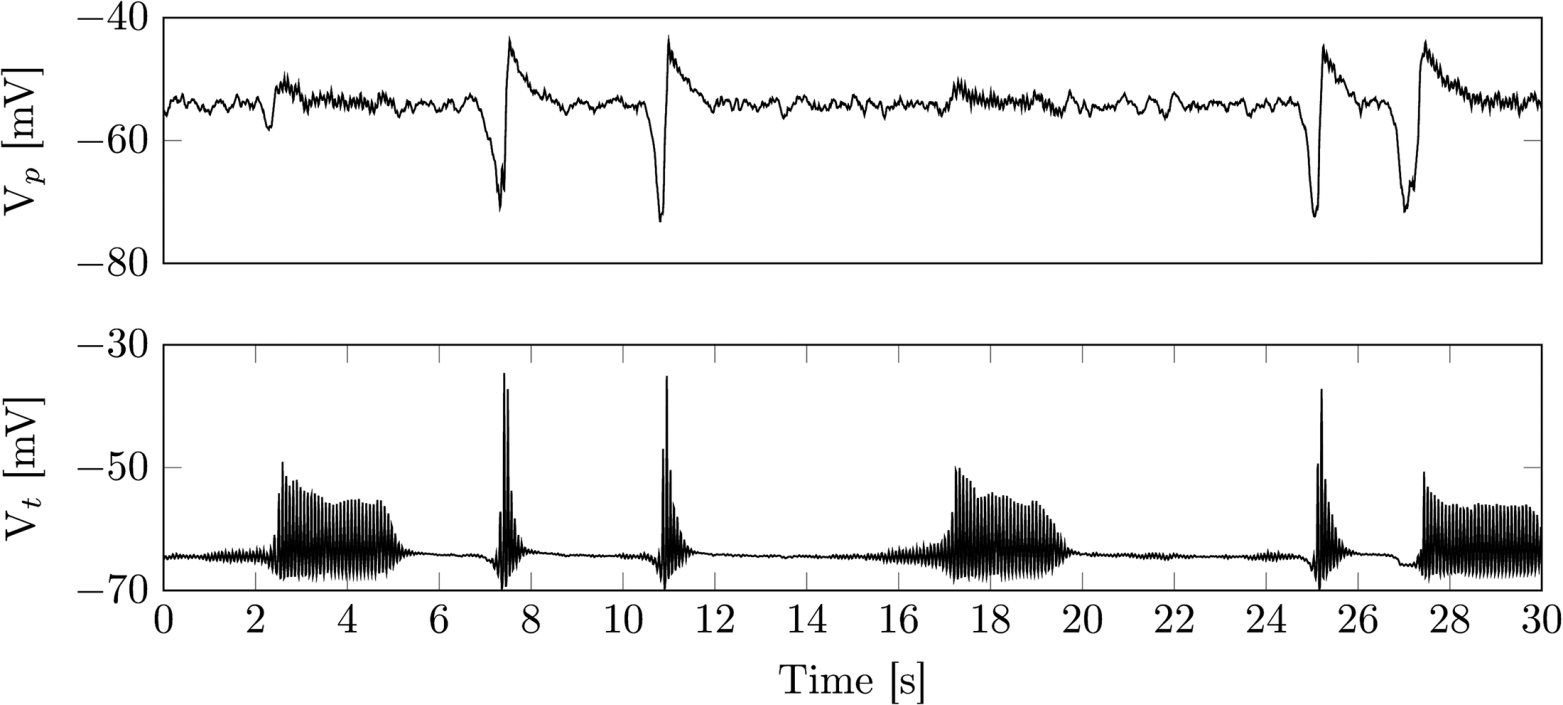
Example time series of sleep stage N2. Shown are membrane voltages of the cortical pyramidal (top) and the thalamic relay population (bottom). The spindle oscillations induced within the thalamic module project into the cortical module. While the spindle oscillations are generally induced by fluctuations in background noise, there is also a grouping between cortical KCs and thalamic spindles (see 7s-9s and 19s-21s). The grouping stems from the lack of depolarizing input during a cortical KC. Parameters are as in Table 2.

As can be seen in Fig 4 spindles may be triggered by KCs in the full model, but may also occur independent of KCs. During a KC the sudden drop of excitatory drive hyperpolarizes the RE and TC population, leading to deinactivation of *I*_T_. The ensuing depolarization upon the transition back to the active state triggers a LTS and a spindle sequence in turn. The spindle then projects back into the depolarizing phase of the KC. This is in good agreement with the grouping of spindles and KCs observed experimentally [6, 74].

Although less likely the model can also give rise to KCs triggered by a spindle. This can be achieved by increasing the connection strength from the thalamic to the cortical module (model output not shown). During N2, KCs occur at a low rate. Hence, spindle initiation and termination are closely linked to the time course of *I*_*h*_ (Fig 3A), similar to the isolated thalamic module. The parameters for the output in Fig 4 are given in Table 2.

Given the parameter setting in Table 2, the cortical module is within a stable focus, close to a Hopf bifurcation accompanied by a canard explosion. This leads to noise driven medium amplitude background oscillations around the stable focus, that are interrupted by large amplitude deflections (KCs). In good agreement with experimental findings, KCs also appear within the isolated cortex, although they may be initiated through thalamic input.

### SOs and spindles during sleep stage N3

On the transition to sleep stage N3 the canard phenomenon vanishes in a cusp bifurcation and only a high amplitude limit cycle remains. SOs are noise driven oscillations around a stable focus, close to a Hopf bifurcation [30].

**Fig 5:**
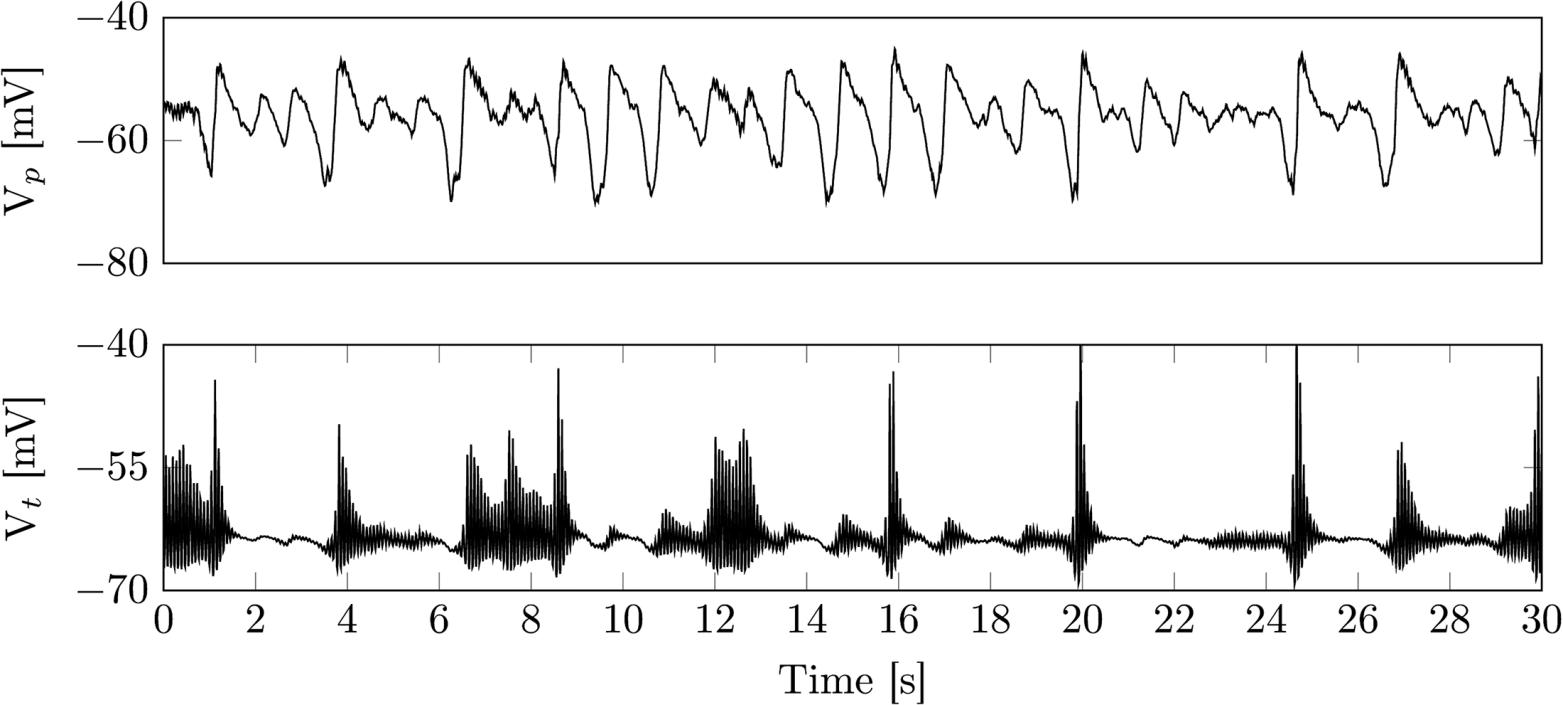
Example time series of sleep stage N3. Shown are membrane voltages of the cortical pyramidal (top) and the thalamic relay population (bottom). During N3 the model shows ongoing slow oscillatory activity. In contrast to sleep stage N2, SOs cannot be identified as isolated events. Furthermore, there are no isolated spindle oscillations and spindle activity is time-locked to SOs. Parameters are given in Table 2.

In contrast to sleep stage N2 spindle initiation and termination are now dominated by the modulatory input from the cortical module, that overrules the *I*_h_ rhythm. Rather than occurring rhythmically spindles are time-locked to the depolarized phase of a SO. In Fig 5 an example time series is shown. Importantly, not every SO is able to trigger a spindle, as can be seen in Fig 5 (9–12 s, 13–15 s). We observed that in a sequence of SOs the first triggers a spindle, which leads to an activation of *I*_h_. This reduces spindle amplitude or even inhibits spindle initiation by the following SO.

### Endogenous event triggered averages

**Fig 6:**
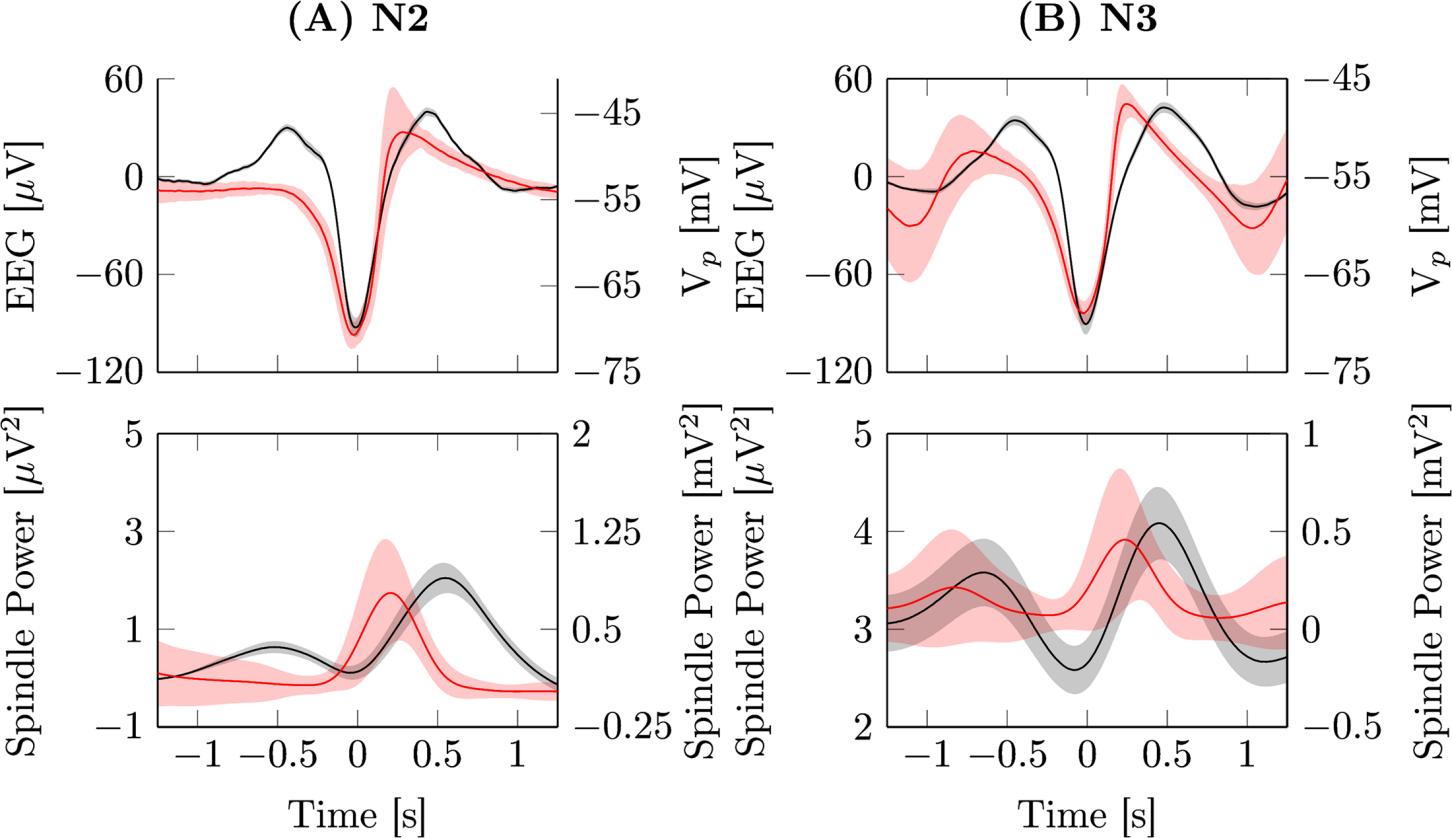
Event triggered average potentials. Averaged EEG signal (top) and fast spindle band power (bottom) time-locked to the negative peaks (t = 0 s) of all detected events from electrode *Cz* (black, left axis) and model output (red, right axis). (A) Detected KCs from data scored as sleep stage N2 (Experiment: 227,45 ± 19,22, Model: 238 events). (B) SO average from data scored as sleep stage N3 (Experiment: 983,64 ± 106,1, Model: 654 events). Each simulation was run for 3600 s with parameters set according to Table 2.

To further validate the model, we determined averages of the generated EEG signal and fast spindle power time-locked to the negative peak of the endogenous KCs/SOs during N2 and N3. This method is often used to illustrate the grouping of spindles by SOs and morphological features of SOs, e.g. in [6, 13, 65]. Model output and data for N2 and N3 is depicted in Fig 6.

As can be seen in Fig 6, the grouping of spindles by SOs is present in the model. Spindle power is highest during the positive half-wave following the negative peak. However, there are some notable differences. Compared to the experimental data the initial depolarization preceding the transition to the down state is less prominent, leading to a shallower slope of the transition to the down state. In the thalamocortical model the transition to the depolarized up state occurs considerably earlier with a time to peak of 300 ms, compared to 440 ms in the data. This stems from strong depolarizing input by thalamic spindle bursts, which start directly after the negative peak of a KC/SO and push the cortex further into the depolarized state. However, this is still in line with other experimental studies, that find different timings of spindles for the supplementary motor area of the cortex [75].

### Closed-loop auditory stimulation

In the following we show the ability of the model to reproduce data from a recent experiment in humans performing auditory closed-loop stimulation during NREM sleep [13]. The stimulation protocol is as follows: After the negative peak of a SO was detected, two auditory stimuli were applied phase-locked to the following positive peak of the depolarized up phase of the detected and the subsequent SO.

In the experimental study the delay time between the negative peak and the ensuing positive half-wave peak was determined for every subject independently. The second stimulus followed after a fixed interval of 1075 ms. Detection was then paused for 2.5 s. We accordingly determined the delay time from the model output, resulting in a delay of 450 ms for the N3 parameter setting. The second stimulus was chosen to occur 1075 ms after the first one and we also paused detection for 2.5 s. Stimuli are given as elevations in mean background noise of the thalamic relay population for a duration of 80 ms.

**Fig 7:**
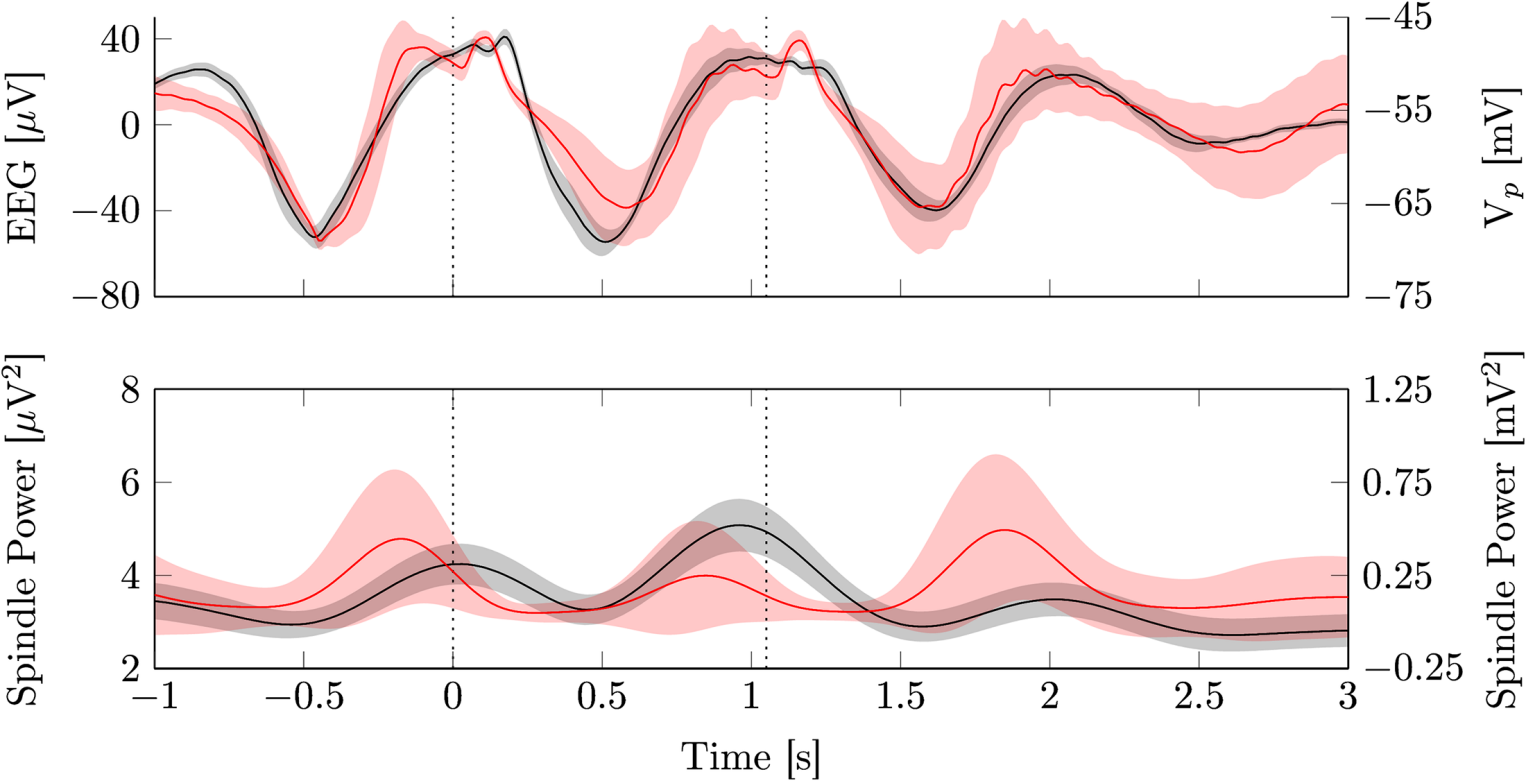
Closed loop stimulation. The upper panel depicts in black the mean (± SEM) evoked potentials of human EEG data from electrode *Cz* during closed loop stimulation, time locked to the first stimulus (11 subjects, 245.6 ± 38.1 stimuli). In red the reproduction of the stimulation protocol with the model is shown (mean ± SD, 88 stimuli). The dashed line marks the stimulus onset. The lower panel shows the corresponding fast spindle power. Parameters used for model simulation are given in Table 2.

Fig 7 shows the averaged EEG signal and model output time-locked to the first stimulus (*t* = 0). There is a good agreement between model output and the experimental data. Especially the large amplitude, late components of the ERP are very close to the original waveform. The early component of the evoked potential, the P200, can be seen in the experimental data after each stimulus, but it is more pronounced in the model output.

In addition, the evoked spindle responses of model and data also have similar time courses. In both cases spindle power is systematically increased during the depolarized up phases induced by the stimuli. However, the strong increase in spindle power seen in the data after the first stimulus is not visible in the model. We hypothesize this to stem from a recruitment effect, where the stimulus activates a larger fraction of the thalamus than the endogenous slow oscillation would. As our thalamic module is a point model without any spatial extent, these effects are excluded by construction.

**Fig 8:**
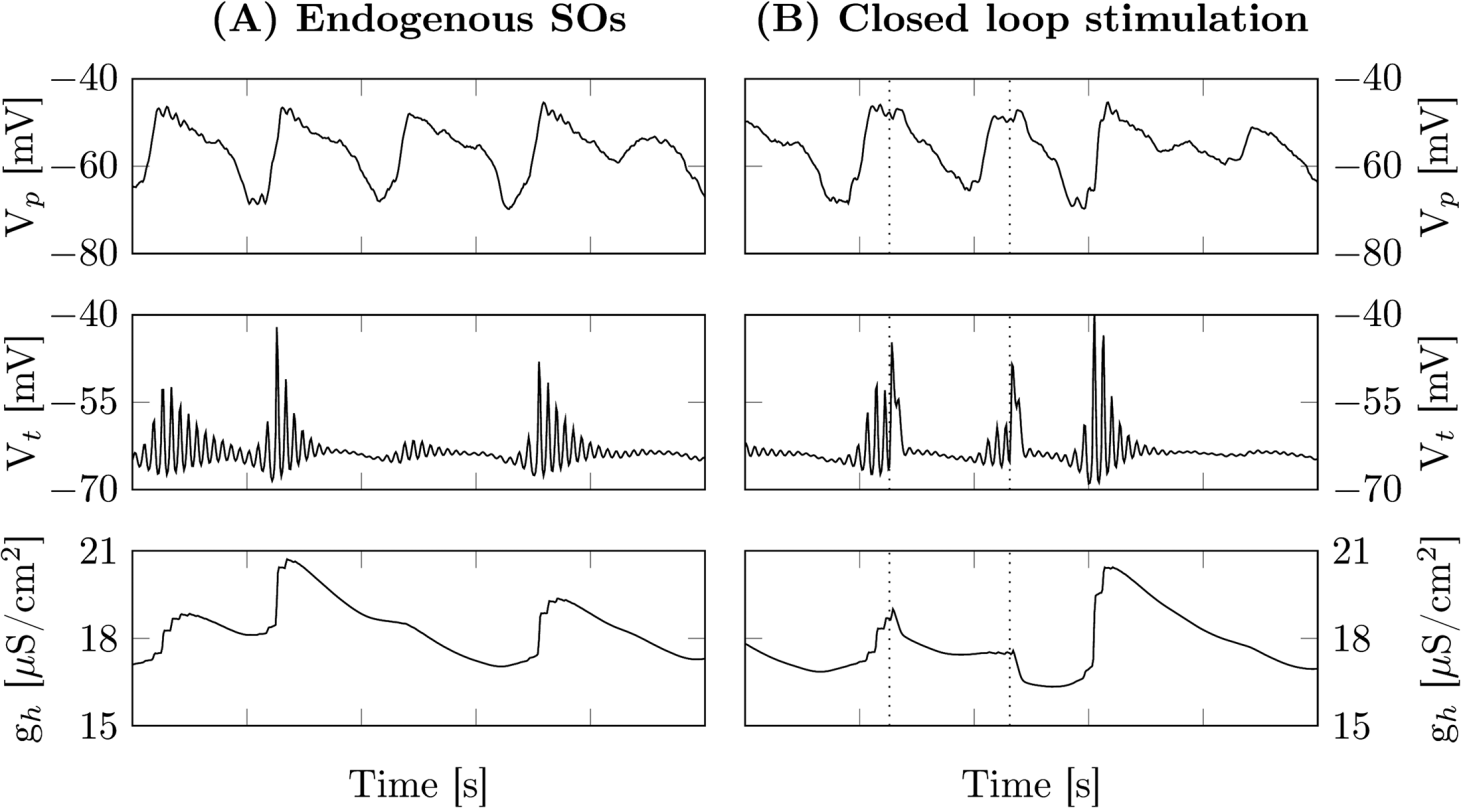
Stimulation disturbs refractoriness. The upper two panels depict the membrane voltages of the pyramidal and thalamic relay populations, respectively. In the third panel the conductivity of the *I*_h_ current is shown. (A) Example time series of an unperturbed train of SOs during sleep stage N3. The first two SOs lead to an activation of *I*_h_, that slowly declines back to baseline levels. As *I*_h_ activation is still well above baseline, the third SO is unable to trigger a spindle response. During the fourth SO *I*_h_ activation is sufficiently low so that a spindle occurs. (B) Shown is an example of closed loop stimulation during sleep stage N3, with the dashed lines indicating stimulus onset. In contrast to the endogenous case, the depolarization of the thalamic relay population induced by the stimulation leads to a rapid decrease in *I*_h_ activation, so that the following SO triggers a spindle. Parameters as in Table 2.

Interestingly, in the experimental data there is a drop in spindle power after the second stimulus is applied. This seems to be a refractoriness of the thalamus after the second slow oscillation, which has also been observed in [76]. Despite the model showing such a refractory period in the isolated thalamus (Fig 3A), as well as during trains of endogenous SOs in the full model (Fig 8A), it lacks it upon stimulation (Fig 8B).

This happens because stimulation disturbs the *I*_h_ mediated spindle termination mechanism. As the stimulation depolarizes the TC population, the calcium concentration drops, because calcium influx through the *I*_T_ current stops and calcium leaks out with a time constant of 10ms. Without the elevated calcium concentration, *I*_h_ deactivates back to baseline levels and immediately allows for a new full fledged spindle.

## Discussion

We developed a neural mass model of the thalamocortical system that produces realistic time courses of EEG signals during sleep stages N2 and N3 and correctly replicates the timing of KCs and spindles. We validated our model with SO triggered averages of the EEG signal and spindle power. Finally, we used the model to reproduce evoked responses from closed-loop auditory stimulation during human NREM sleep.

### Mechanisms of spindle generation

The model emphasizes the role of *I*_T_ and *I*_h_ currents in the generation of thalamocortical rhythms as they were sufficient to reproduce the investigated EEG phenomena. It reproduces the grouping of spindles and KCs/SOs, observed in human EEG [6], that is thought to play a crucial role in the consolidation of memory [77, 78]. Additionally, it exhibits refractoriness of spindle oscillations, i.e. not every SO in a train of endogenous SOs triggers a spindle. Although adding extra currents increases dimensionality and parameter space, the model still preserves the overall simplicity and computational efficacy common to neural mass models.

### Spindle timing

Relative to the negative deflection of a KC, spindles consistently start earlier than in the data. Consistently, the depolarizing up phase of endogenous KCs and SOs arrives earlier in the model than in the data. A comparison with the results from the isolated cortical module shows, that this is mostly due to strong depolarizing input from the thalamus. Yet, there is no clear explanation for the difference between model and experiment. It might be due to the simplification of the intrinsic mechanisms, e.g. firing rate adaptation in cortex and spindle dynamics in thalamus. On the other hand it could also be that finer details, e.g. spatial extension or the layered structure of the cortex are important for its temporal dynamics. Also the way conduction delays between cortex and thalamus were implemented, namely via an extra convolution with an alpha function, might play a role.

### Auditory stimulation

A recent experimental study suggests that the refractoriness of thalamic spindles is a limiting factor for the impact of auditory stimulation upon memory consolidation [76]. They found, that longer trains of stimuli do *not* provide any benefit in memory consolidation compared to the two stimulus protocol. Remarkably, the first stimulus triggers a strong spindle, whereas the following stimuli show a diminished spindle response. This clearly indicates the importance of the grouping of spindles and SOs for the consolidation of memory. In contrast to these experimental findings, auditory stimulation in the model alleviates the refractoriness of the thalamic module, leading to spindle oscillations with similar amplitude following every stimulus. This is because strong depolarization of the thalamic populations by the stimulus interrupts the thalamic *I*_h_ rhythm. We see this as a challenge for the understanding of how auditory stimulation is processed during sleep and how it interacts with spindle generation.

### Relation to other work

Recently, Cona et al. also developed a neural mass model to describe the sleeping thalamocortical system [79]. They combined two distinct firing modes via the activation of the T-type calcium current, showing that this multiplicative change in firing rate can lead to periodic spindle-like oscillations. However, in this study we include the currents directly into the equation of the membrane voltage, similar to [30, 54]. Our model relates directly to scalp EEG signals during natural sleep and auditory stimulation.

### Effect of neuromodulators and sleep regulation

In our model, we induce the transition between the different sleep stages by changes of the three key parameters (*g*_KNa_ and *σ*_*p*_ in the cortex and ${\bar{g}}_{\text{LK}}$ in the thalamus), that are directly linked to the action of neuromodulators [30, 80–82]. These parameters are known to be affected by neuromodulators, such as noradrenalin, serotonin and acetylcholine [44, 46, 50, 51, 83], whose concentrations vary over the night. Regulation of neuromodulator concentrations arises through complex interactions within different sleep regulatory networks [53, 84]. Recently there has been progress in the mathematical description of sleep regulatory networks [85–89]. However, as we focus on the different dynamical modes the thalamocortical system can exhibit and how thalamus and cortex interact, we do not include sleep regulation in this manuscript.

### Are KCs biphasic or triphasic?

The waveform of a KC has been described as being biphasic, consisting of a large negative deflection (down state) followed by a pronounced depolarization (up state)—or triphasic, comprising an initial positive bump followed by a down state and an up state. Menicucci et al. [90] analyzed the shapes of KCs in N2 and N3 and found that on average a triphasic pattern, up-down-up, is present in both sleep stages. Our model does not show this sequence for sleep stage N2. *In vivo*, sleep stage N2 is rarely stationary and spans varying depths of sleep, as well as transitions to other sleep stages. In contrast, our model depicts idealized N2 at a single point in time, to separate it from wakefulness and N3. Choosing a parameter setting closer to N3 will naturally give rise to a depolarization preceding the down state. We predict that biphasic KCs should be found mostly in early N2 or very late N2, as in the second half of the night after the major SWS episodes.

### Is there true bistability during natural sleep?

The model is consistent with the observation that during N2 and N3 of natural sleep the cortex is mostly in the active state [91]. We adopt the view of [30], where KCs were characterized as transient events—reversed spikes—initiated by a canard explosion. Consequently the down state is never stable in our model. This may seem counterintuitive as many intracellular recordings support the notion of bistability. However, neural mass models represent population averages, whereas intracellular recordings only sample individual members of a population, leaving open this alternative interpretation derived from stereotypical graphoelements in the EEG.

## Supporting Information

S1 Text Model equations. This section provides the full mathematical description of the model presented here.
(PDF)

S2 Text Approximation of long range connection delay. This section provides a justification of the approximation of the thalamocortical transmission delay by a convolution with an alpha function.
(PDF)

S1 Table Parameter values. This table defines all constants used throughout this paper.
(PDF)

S1 Dataset Experimental data. This dataset includes the experimental data used for Figs 6 and 7. Given are the time-locked averages for every subject. In Fig 6 only the sham condition was used to represent endogenous KCs/SOs, whereas Fig 7 displays the stimulation condition. For detailed information on data acquisition and processing please see the original study.
(XLS)
