# Supplementary material for: A Thalamocortical Neural Mass Model of the EEG during NREM Sleep and Its Response to Auditory Stimulation
Source: PLoS Comput Biol. 2016 Sep 1;12(9):e1005022. doi: 10.1371/journal.pcbi.1005022 (PMC5008627; doi:10.1371/journal.pcbi.1005022)
Supplement: S1 Text — This section provides the full mathematical description of the model presented here. (PDF) [file pcbi.1005022.s001.pdf]

Supporting Information

Text S1 Model equations

The complete mathematical description of the full thalamocortical model:

$$\begin{aligned}
\tau_p \dot{V}_p &= -J_{\mathrm{L}}^p - J_{\mathrm{AMPA}}(s_{ep}) - J_{\mathrm{GABA}}(s_{gp}) - C_m^{-1}\tau_p I_{\mathrm{KNa}} \\
\tau_i \dot{V}_i &= -J_{\mathrm{L}}^k - J_{\mathrm{AMPA}}(s_{ei}) - J_{\mathrm{GABA}}(s_{gi}) \\
\tau_t \dot{V}_t &= -J_{\mathrm{L}}^t - J_{\mathrm{AMPA}}(s_{et}) - J_{\mathrm{GABA}}(s_{rt}) - C_m^{-1}\tau_t (I_{\mathrm{LK}}^t - I_{\mathrm{T}}^t - I_{\mathrm{h}}) \\
\tau_r \dot{V}_r &= -J_{\mathrm{L}}^r - J_{\mathrm{AMPA}}(s_{er}) - J_{\mathrm{GABA}}(s_{rr}) - C_m^{-1}\tau_r (I_{\mathrm{LK}}^r - I_{\mathrm{T}}^r) \\
\ddot{s}_{ep} &= \gamma_e^2 \left(N_{pp} Q_p(V_p) + N_{pt}\phi_t + \phi_n - s_{ep}\right) - 2\gamma_e \dot{s}_{ep} \\
\ddot{s}_{ei} &= \gamma_e^2 \left(N_{ip} Q_p(V_p) + N_{it}\phi_t + \phi_n^{'} - s_{ei}\right) - 2\gamma_e \dot{s}_{ei} \\
\ddot{s}_{et} &= \gamma_e^2 \left(N_{tp}\phi_p + \phi_n^{''} - s_{et}\right) - 2\gamma_e \dot{s}_{et} \\
\ddot{s}_{er} &= \gamma_e^2 \left(N_{rt} Q_t(V_t) + N_{rp}\phi_p - s_{er}\right) - 2\gamma_e \dot{s}_{er} \\
\ddot{s}_{gp} &= \gamma_g^2 \left(N_{pi} Q_i(V_i) - s_{gp}\right) - 2\gamma_g \dot{s}_{gp} \\
\ddot{s}_{gi} &= \gamma_g^2 \left(N_{ii} Q_i(V_i) - s_{gi}\right) - 2\gamma_g \dot{s}_{gi} \\
\ddot{s}_{rt} &= \gamma_r^2 \left(N_{tr} Q_r(V_r) - s_{it}\right) - 2\gamma_r \dot{s}_{rt} \\
\ddot{s}_{rr} &= \gamma_r^2 \left(N_{rr} Q_r(V_r) - s_{rr}\right) - 2\gamma_r \dot{s}_{rr} \\
\ddot{\phi}_p &= \nu^2 \left(Q_p(V_p) - \phi_p\right) - 2\nu \dot{\phi}_p \\
\ddot{\phi}_t &= \nu^2 \left(Q_t(V_t) - \phi_t\right) - 2\nu \dot{\phi}_t \\
\dot{h}_{\mathrm{T}}^t &= (h_\infty^t - h_{\mathrm{T}}^t)/\tau_{\mathrm{h}}^t \\
\dot{h}_{\mathrm{T}}^r &= (h_\infty^r - h_{\mathrm{T}}^r)/\tau_{\mathrm{h}}^r \\
\dot{m}_{\mathrm{h1}} &= (m_\infty^h (1 - m_{\mathrm{h2}}) - m_{\mathrm{h1}})/\tau_m^{\mathrm{h}} - k_3 P_{\mathrm{h}} m_{\mathrm{h1}} + k_4 m_{\mathrm{h2}} \\
\dot{m}_{\mathrm{h2}} &= k_3 P_{\mathrm{h}} m_{\mathrm{h1}} - k_4 m_{\mathrm{h2}} \\
[\dot{\mathrm{Ca}}] &= \alpha_{\mathrm{Ca}} I_{\mathrm{T}}^t - ([\mathrm{Ca}] - \mathrm{Ca}_0)/\tau_{\mathrm{Ca}} \\
[\dot{\mathrm{Na}}] &= (\alpha_{\mathrm{Na}} Q_p(V_p) - \mathrm{Na}_{\mathrm{pump}}([\mathrm{Na}]))/\tau_{\mathrm{Na}}.
\end{aligned} \tag{A.1}$$

The currents are given by the following equations:

$$\begin{aligned}
J_{\mathrm{L}}^k &= (V_i - E_{\mathrm{L}}^k) \\
J_{\mathrm{AMPA}}(s_{ek}) &= w_{\mathrm{AMPA}} s_{ek} (V_k - E_{\mathrm{AMPA}}) \\
J_{\mathrm{GABA}}(s_{gk}) &= w_{\mathrm{GABA}} s_{gk} (V_k - E_{\mathrm{GABA}}) \\
J_{\mathrm{GABA}}(s_{rk}) &= w_{\mathrm{GABA}} s_{rk} (V_k - E_{\mathrm{GABA}}) \\
I_{\mathrm{LK}}^k &= \bar{g}_{\mathrm{LK}} (V_i - E_{\mathrm{K}}^k) \\
I_{\mathrm{T}}^t &= \bar{g}_{\mathrm{T}}^t m_\infty^t m_\infty^t h^t (V_t - E_{\mathrm{Ca}}) \\
I_{\mathrm{T}}^r &= \bar{g}_{\mathrm{T}}^r m_\infty^r m_\infty^t h^r (V_r - E_{\mathrm{Ca}}) \\
I_{\mathrm{h}} &= \bar{g}_{\mathrm{h}} (m_{\mathrm{h1}} + g_{inc} m_{\mathrm{h2}})(V_t - E_{\mathrm{h}}) \\
I_{\mathrm{KNa}} &= \bar{g}_{\mathrm{KNa}} \frac{0.37}{1 + \left(\frac{38.7}{[\mathrm{Na}]}\right)^{3.5}} (V_p - E_{\mathrm{K}})
\end{aligned} \tag{A.2}$$

Gating functions are taken as

$$\begin{aligned}
m_\infty^t &= \frac{1}{1+\exp(-(V_t+59)/6.2))} \\
m_\infty^r &= \frac{1}{1+\exp(-(V_r+52)/7.4))} \\
h_\infty^t &= \frac{1}{1+\exp((V_t+81)/4))} \\
h_\infty^r &= \frac{1}{1+\exp((V_r+80)/5))} \\
\tau_\mathrm{h}^t &= (30.8+(211.4+\exp((V_t+115.2)/5))/(1+\exp((V_t+86)/3.2)))/3^{1.2} \\
\tau_\mathrm{h}^r &= (85+1/(\exp((V_r+48)/4)+\exp(-(V_r+407)/50)))/3^{1.2} \\
m_\infty^h &= \frac{1}{1+\exp((V_t+75)/5.5))} \\
\tau_m^\mathrm{h} &= (20+1000/(\exp((V_t+71.5)/14.2)+\exp(-(V_t+89)/11.6))) \\
P_\mathrm{h} &= k_1[\mathrm{Ca}]^{n_P}/(k_1[\mathrm{Ca}]^{n_P}+k_2)
\end{aligned} \tag{A.3}$$

The sodium pump is given by

$$\mathrm{Na}_\mathrm{pump}([\mathrm{Na}]) = R_\mathrm{pump}\left(\frac{[\mathrm{Na}]^3}{[\mathrm{Na}]^3+3375} - \frac{[\mathrm{Na}_0]^3}{[\mathrm{Na}_0]^3+3375}\right) \tag{A.4}$$

Finally the firing rate function obeys

$$Q_k = \frac{Q_k^\mathrm{max}}{1+\exp(-(V_k-\theta)/\sigma_k)} \tag{A.5}$$
