# Supplementary material for: A Thalamocortical Neural Mass Model of the EEG during NREM Sleep and Its Response to Auditory Stimulation
Source: PLoS Comput Biol. 2016 Sep 1;12(9):e1005022. doi: 10.1371/journal.pcbi.1005022 (PMC5008627; doi:10.1371/journal.pcbi.1005022)
Supplement: S2 Text — This section provides a justification of the approximation of the thalamocortical transmission delay by a convolution with an alpha function. (PDF) [file pcbi.1005022.s002.pdf]

Supporting Information

Text S2 Approximation of long range connection delay

As discussed in the methods section (Eq. 11), long range connections are modeled by a convolution with an alpha function representing the average axonal conduction delay. This is an approximation of the delay differential equations that describe the axonal conduction delay, which we justify briefly.

First, the alpha function acts as a lowpass filter with magnitude response function $|H(\omega)| = \nu^2/(\nu^2 + \omega^2)$ and cutoff frequency $f_c = \nu/(2\pi)$. Hence, with $\nu = 120 \cdot 10^{-3}$ ms^{-1} the input is attenuated by 3dB at ≈ 19 Hz. This is not problematic as long as the model does not generate sharp discontinuities or high frequency oscillations, which is the case for our model (see Fig. S2.1).

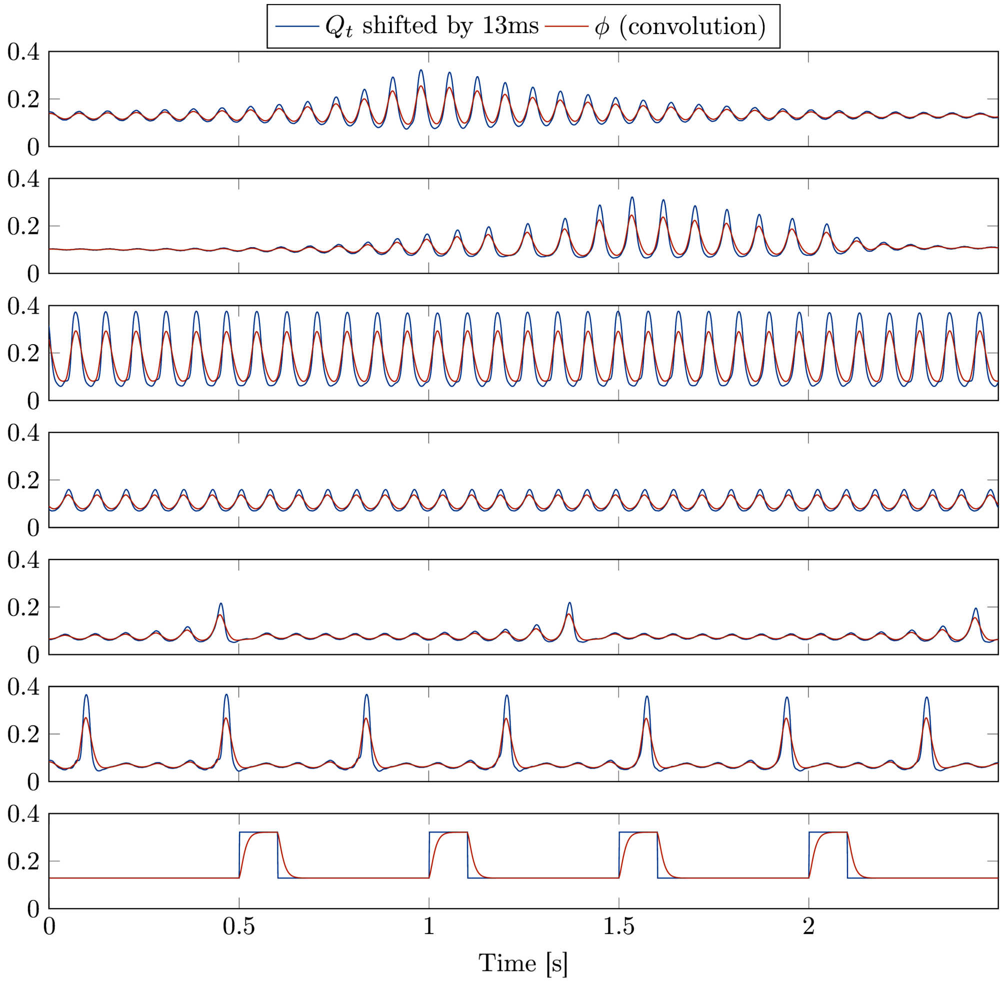

Figure S2.1. Comparison of convolved and time shifted signals. The panels depict a comparison of the thalamic firing rate time shifted by 13 ms and the axonal flux ϕ. The upper 6 panels represent excerpts from the time series shown in Fig. 3, with a focus on the sharpest features. The bottom panel additionally compares both signals for a delta pulse.

Second, physiological measurements suggest that the transmission delay between the thalamus and the cortex is in the range of a few milliseconds [1–4], in particular for neurons receiving sensory inputs. Recent investigations in humans find a one-way conduction delay in the range of 12.5-19.8 ms [5]. We find that for those cases the effective delay is well approximated by the median of the alpha function $\Delta t = -1 - W_{-1}(-\frac{1}{2e}) = 1.67835/\nu$, where W_{-1} is the bottom branch of the Lambert W function (see Fig. S2.2). Please note that the effective delay is considerably larger than the time to peak of the alpha function. This is due to the asymmetric shape of the alpha function, which has a heavy tail.

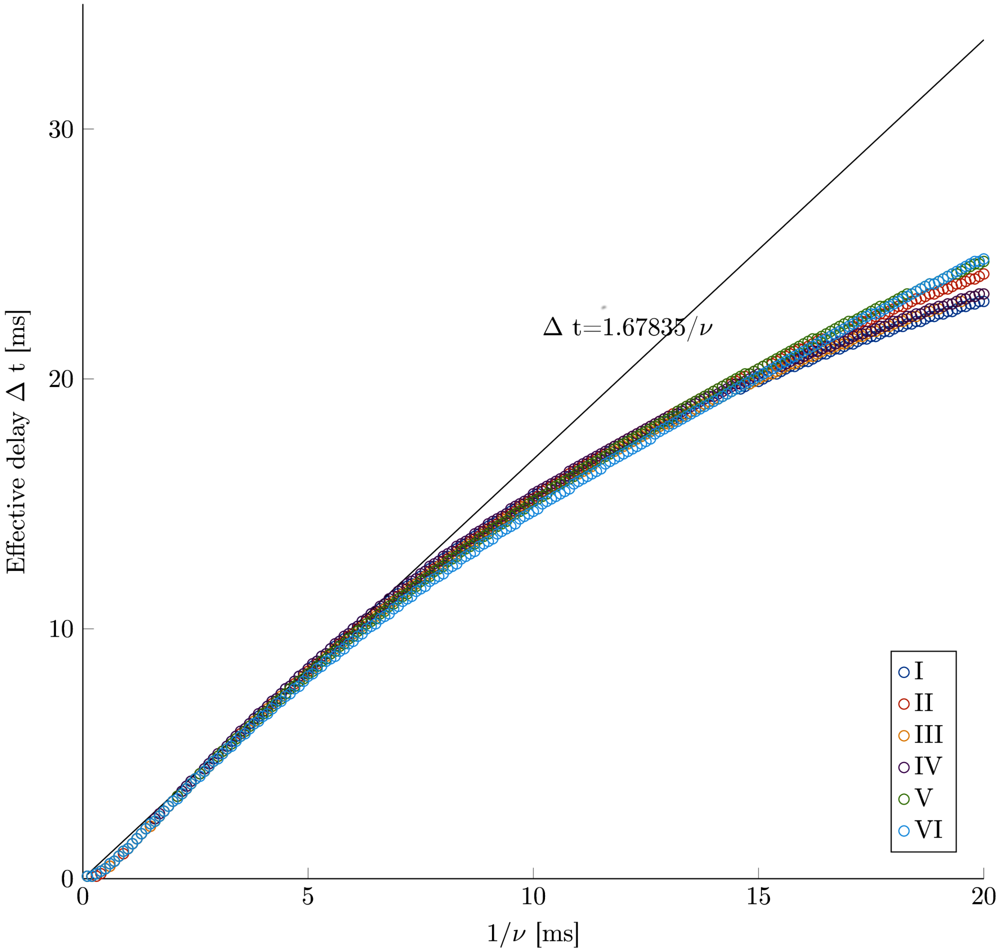

Figure S2.2. Effective delay for different rate constants. Here the effective delay determined by crosscorrelation of the different time series from Fig. 3 is depicted for a range of axonal rate constants ν. For comparison the median of the respective alpha function is depicted in black.

References

1. Agmon A, Connors BW. Correlation between intrinsic firing patterns and thalamocortical synaptic responses of neurons in mouse barrel cortex [Journal Article]. The Journal of Neuroscience. 1992;12(1):319–329.

2. Swadlow HA. Information flow along neocortical axons. In: Time and the Brain. Conceptual Advances in Brain Research. Amsterdam: Harwood Academic Publishers; 2000. p. 150–179.

3. Salami M, Itami C, Tsumoto T, Kimura F. Change of conduction velocity by regional myelination yields constant latency irrespective of distance between thalamus and cortex. Proceedings of the National Academy of Sciences of the United States of America. 2003;100(10):6174–9.

4. Gentet LJ, Ulrich D. Electrophysiological characterization of synaptic connections between layer VI cortical cells and neurons of the nucleus reticularis thalami in juvenile rats [Journal Article]. European Journal of Neuroscience. 2004;19(3):625–633.

5. Roux F, Wibral M, Singer W, Aru J, Uhlhaas PJ. The phase of thalamic alpha activity modulates cortical gamma-band activity: evidence from resting-state MEG recordings. The Journal of Neuroscience. 2013;33(45):17827–17835.
