# Supplementary material for: A Thalamocortical Neural Mass Model of the EEG during NREM Sleep and Its Response to Auditory Stimulation
Source: PLoS Comput Biol. 2016 Sep 1;12(9):e1005022. doi: 10.1371/journal.pcbi.1005022 (PMC5008627; doi:10.1371/journal.pcbi.1005022)
Supplement: S1 Table — This table defines all constants used throughout this paper. (PDF) [file pcbi.1005022.s003.pdf]

# Supporting Information Tables

## Table S1 Parameters

This table describes all symbols used within the model.

**Table 1. Symbol description**

| | |
|---|---|
| $C_m$ | Membrane capacitance in the HH model |
| $Q_k^{\max}$ | Maximal firing rate of population $k$ |
| $\theta_k$ | Firing threshold of population $k$ (half activation) |
| $\sigma_k$ | Default gain coefficient of the firing rate function of population $k$ (inverse neural gain) |
| $\tau_k$ | Membrane time constant of population $k$ |
| $\gamma_m$ | Synaptic rate constant of synapse type $m$ |
| $\nu$ | Axonal rate constant |
| $N_{kl}$ | Connectivity constant from presynaptic population $l$ to postsynaptic population $k$ |
| $w_X$ | Input rate of synaptic channel of type $X$ |
| $\bar{g}_X$ | Conductivity of ion channel $X$ |
| $E_X$ | Nernst reversal potential of channel $X$ |
| $\alpha_{\mathrm{Na}}$ | Sodium influx through firing rate |
| $\tau_{\mathrm{Na}}$ | Time constant of sodium extrusion |
| $R_{\mathrm{pump}}$ | Strength of the sodium pump |
| $\mathrm{Na}_{\mathrm{eq}}$ | Resting state sodium equilibrium |
| $\alpha_{\mathrm{Ca}}$ | Calcium influx rate |
| $\tau_{\mathrm{Ca}}$ | Calcium time constant |
| $\mathrm{Ca}_0$ | Calcium resting state concentration |
| $k_j$ | Reaction velocity of h-current |
| $n_P$ | Number of calcium binding sites |
| $g_{inc}$ | Conductivity scaling of h-current |
| $\phi_0$ | Mean background noise |
| $\phi_C^{\mathrm{sd}}$ | Standard deviation of cortical background noise |
| $\phi_T^{\mathrm{sd}}$ | Standard deviation of thalamic background noise |

**Table 2. Parameter values**

| Symbol | Value | Unit |
|---|---|---|
| $C_m$ | 1 | $\mu$F/cm$^2$ |
| $\tau_p, \tau_i$ | 30 | ms |
| $\tau_t, \tau_r$ | 20 | ms |
| $Q_p^{\max}$ | $30{\cdot}10^{-3}$ | ms$^{-1}$ |
| $Q_i^{\max}$ | $60{\cdot}10^{-3}$ | ms$^{-1}$ |
| $Q_t^{\max}$, $Q_r^{\max}$ | $400{\cdot}10^{-3}$ | ms$^{-1}$ |
| $\theta$ | $-58.5$ | mV |
| $\sigma_p$ | 4 | mV |
| $\sigma_i$, $\sigma_t$, $\sigma_r$ | 6 | mV |
| $\gamma_e$ | $70{\cdot}10^{-3}$ | ms$^{-1}$ |
| $\gamma_g$ | $58.6{\cdot}10^{-3}$ | ms$^{-1}$ |
| $\gamma_r$ | $100{\cdot}10^{-3}$ | ms$^{-1}$ |
| $\nu$ | $120{\cdot}10^{-3}$ | ms$^{-1}$ |
| $N_{pp}$ | 120 | - |
| $N_{ip}$ | 72 | - |
| $N_{pi}$, $N_{ii}$ | 90 | - |
| $N_{tp}$, $N_{rp}$ | 2.6 | - |
| $N_{rt}$ | 3 | - |
| $N_{tr}$ | 5 | - |
| $N_{rr}$ | 19 | - |
| $N_{pt}$, $N_{it}$ | 2.5 | - |
| $w_{\text{AMPA}}$, $w_{\text{GABA}}$ | 1 | ms |
| $\bar{g}_{\text{T}}^t$ | 3 | mS/cm$^2$ |
| $\bar{g}_{\text{T}}^r$ | 2.3 | mS/cm$^2$ |
| $\bar{g}_{\text{KNa}}$ | 1.33 | mS/cm$^2$ |
| $E_{\text{L}}^p$, $E_{\text{L}}^i$ | $-64$ | mV |
| $E_{\text{L}}^t$, $E_{\text{L}}^r$ | $-70$ | mV |
| $E_{\text{K}}$ | $-100$ | mV |
| $E_{\text{Ca}}$ | 120 | mV |
| $E_{\text{Ca}}$ | $-40$ | mV |
| $E_{\text{AMPA}}$ | 0 | mV |
| $E_{\text{GABA}}$ | $-70$ | mV |
| $\alpha_{\text{Na}}$ | 2 | mM/mA ms |
| $\tau_{\text{Na}}$ | 1.7 | ms |
| $R_{\text{pump}}$ | 0.09 | mM ms$^{-1}$ |
| $\text{Na}_{\text{eq}}$ | 9.5 | mM |
| $\alpha_{\text{Ca}}$ | $51.8{\cdot}10^{-6}$ | mM/mA ms |
| $\tau_{\text{Ca}}$ | 10 | ms |
| $\text{Ca}_0$ | $2.4{\cdot}10^{-4}$ | mM |
| $k_2$ | $4{\cdot}10^{-4}$ | ms$^{-1}$ |
| $k_3$ | $1{\cdot}10^{-1}$ | ms$^{-1}$ |
| $k_4$ | $1{\cdot}10^{-3}$ | ms$^{-1}$ |
| $n_P$ | 4 | - |
| $g_{inc}$ | 2 | - |
| $\phi_0$ | 0 | ms$^{-1}$ |
| $\phi_C^{\text{sd}}$ | $120{\cdot}10^{-3}$ | ms$^{-1}$ |
| $\phi_T^{\text{sd}}$ | $10{\cdot}10^{-3}$ | ms$^{-1}$ |
